# p53 Family: Role of Protein Isoforms in Human Cancer

**DOI:** 10.1155/2012/687359

**Published:** 2011-10-09

**Authors:** Jinxiong Wei, Elena Zaika, Alexander Zaika

**Affiliations:** Department of Surgery and Cancer Biology, Vanderbilt University Medical Center, 1255 Light Hall, 2215 Garland Avenue, Nashville, TN 37232, USA

## Abstract

*TP53*, *TP63*, and *TP73* genes comprise the p53 family. Each gene produces protein isoforms through multiple mechanisms including extensive alternative mRNA splicing. Accumulating evidence shows that these isoforms play a critical role in the regulation of many biological processes in normal cells. Their abnormal expression contributes to tumorigenesis and has a profound effect on tumor response to curative therapy. This paper is an overview of isoform diversity in the p53 family and its role in cancer.

## 1. Introduction

Alternative splicing allows a single gene to express multiple protein variants. It is estimated that 92–95% of human multiexon genes undergo alternative splicing [1, 2]. Abnormal alterations of splicing may interfere with normal cellular homeostasis and lead to cancer development [3–5]. 

 The p53 protein family is comprised of three transcription factors: p53, p63, and p73. Phylogenetic analysis revealed that this family originated from a p63/73-like ancestral gene early in metazoan evolution [6, 7]. Maintenance of genetic stability of germ cells seems to be its ancestral function [8]. The p53 family regulates many vital biological processes, including cell differentiation, proliferation, and cell death/apoptosis [9, 10]. Dysregulation of the p53 family plays a critical role in tumorigenesis and significantly affects tumor response to therapy. This review summarizes current data on the regulation of p53, p63, and p73 isoforms and their roles in cancer.

## 2. Structure and Function

**Figure 1:**
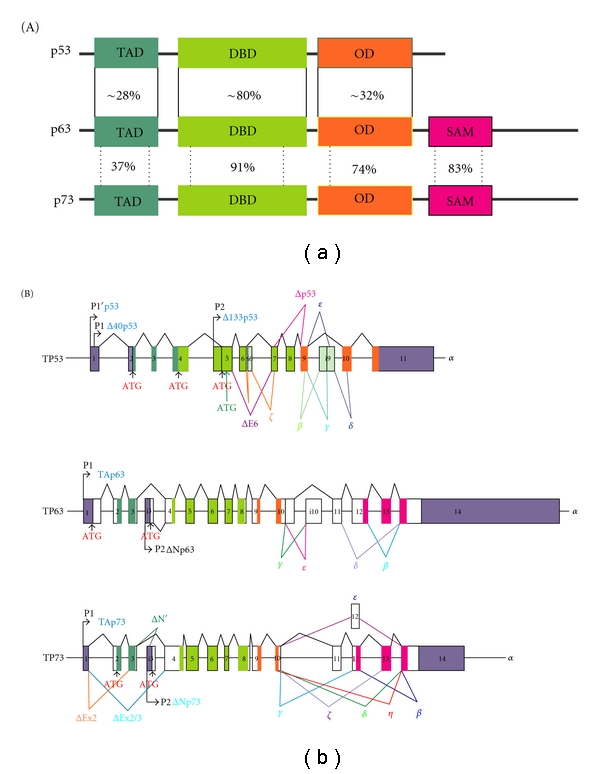
Architectures of human *TP53, TP73, *and* TP63* genes. (A) *TP53, TP73, *and* TP63* genes encode the transactivation (TAD), DNA-binding (DBD), and oligomerization (OD) domains. *TP73* and *TP63* encode additional SAM (Sterile Alpha Motif) domain. Percentage homology of residues between p53, p63, and p73 is shown [11]. (B)* TP53, TP63, *and* TP73* genes have two promoters (P1 and P2). The P1 promoters produce transactivation-competent full-length proteins (TA) while the P2 promoters produce TAD-deficient proteins (ΔN) with dominant-negative functions. p53 gene transcription is initiated from two distinct sites (P1 and P1′).

p53, p63, and p73 genes are located on chromosomes 17p13.1, 3q27-29, and 1p36.2-3, respectively. These genes encode proteins with similar domain structures and significant amino acid sequence homology in the transactivation, DNA-binding and oligomerization domains (Figure 1). The highest amino acid identity is in the DNA-binding domain (~60%). Evolutionally, this domain is the most conserved, suggesting that regulation of transcription plays a pivotal role in an array of functions attributed to the p53 family. Less similarity is found in the oligomerization and transactivation domains (~30%).

 The founding member of the p53 family, the p53 protein, had been discovered more than three decades ago [12, 13]. For a long time, it had been assumed that p53 is expressed as a single polypeptide. However, when it had been found that the p63 and p73 genes encoded a large variety of diverse transcripts, the p53 gene transcription was revisited. Now we know that p53 forms multiple variants. 

 Transcriptions of p53, p63, and p73 genes are regulated by similar mechanisms. It is controlled by two promoters: P1 and P2, where P2 is an alternative intragenic promoter (Figure 1). One study *in silico* provided evidence for the existence of a third putative promoter in the first intron of human *TP73* gene [14]. Therefore, it would not be surprising if additional gene promoters will be found in the future. An extensive alternative splicing adds further diversity to the promoters' products. The produced transcripts and proteins can be generally categorized into two main groups, termed TA and ΔN [15, 16]. TA variants contain the N-terminal transactivation domain while ΔN isoforms lack the entire (or part of) domain. It was initially thought that ΔN isoforms are only generated by the P2 promoter whereas the P1 promoter regulates TA isoforms. Further analysis of alternative mRNA splicing revealed that some transcriptionally deficient isoforms are products of the P1 promoter. For example, the P1 promoter of the *TP73* gene regulates TAp73 isoforms and isoforms, which lack the TA domain: ΔEx2p73, ΔEx2/3p73, and ΔN′p73. The latter isoforms are missing either exon 2 (ΔEx2p73) or both exon 2 and 3 (ΔEx2/3p73) or contain an additional exon 3′ (ΔN′p73) [17, 18]. Other ΔNp73 transcripts are products of the P2 promoter. Similar to p73, the P1 promoter of the p53 gene produces transcriptionally active isoforms [5]. The alternative splicing is responsible for transcriptionally deficient isoforms of Δ40p53, which missing the first 40 amino acids at the N-terminus [5, 19, 20]. Additional p53 transcriptionally deficient isoforms (Δ133p53 and Δ160p53) are regulated by the P2 promoter located in intron 4 of the p53 gene [5, 21]. 

 Additional diversity of p53, p63, and p73 transcripts is generated by alternative splicing at the 3′ end of the transcripts (Figure 1). These splice variants are traditionally named with letters of the Greek alphabet. Initially, three such splice variants have been described for p63 and p53 (*α*, *β*, *γ*), and nine for p73 (*α*, *β*, *γ*, *δ*, *ε*, *θ*, *ζ*, *η*, and *η*1) [22–25]. Later, additional p63 splice variants (*δ*, *ε*) and p53 (*δ*, *ε*, *ζ*, ΔE6) were reported [26–28]. However, it should be noted that a majority of p53, p63, and p73 studies focus on a few isoforms, primarily *α*, *β*, and *γ*. Little is known about the functions of other isoforms. The combination of alternative splicing at the 5′ and 3′ ends, alternative initiation of translation and alternative promoter usage can significantly increase protein diversity. For example, N-terminal variants (p53, Δ40p53, Δ133p53, and Δ160p53) can be produced in *α*, *β*, and *γ* “flavors” [20, 21]. Theoretically, the p53 gene can produce at least 20 isoforms, p63 at least 10, and p73 more than 40, though not all have been experimentally confirmed. 

 p53, TAp63, and TAp73 share significant functional resemblance. They can induce cell cycle arrest, apoptosis, or cellular senescence. This similarity can be explained, at least in part, by transactivation of the same transcriptional targets. Genome-wide analyses found an overlap of the transcription profiles of p53, TAp73, and TAp63, though unique targets were identified as well. Analyses using chromatin immunoprecipitation, reporter, and gel-shift assays found that TAp73 and TAp63 interact with p53-responsive elements. 

 The transactivation and apoptotic potential of p53, TAp73, and TAp63 vary greatly depending on the isoform. TAp63*γ* and TAp73*β* are similar to that of p53*α* [29]. Other isoforms are considered less active on the p53 target gene promoters [9, 23, 30]. Some isoforms are characterized by a variation in domain structure. TAp73*α* and TAp63*α* have an additional domain at the COOH-terminus that is not found in p53. This domain, termed SAM or Sterile Alpha Motif, is responsible for protein-protein interactions and is found in a diverse range of proteins that are involved in developmental regulation. It is also implicated in transcriptional repression [31]. Beta and gamma isoforms of p53 are missing most of the oligomerization domain that results in decreased transcriptional activity [5, 32, 33].

**Figure 2:**
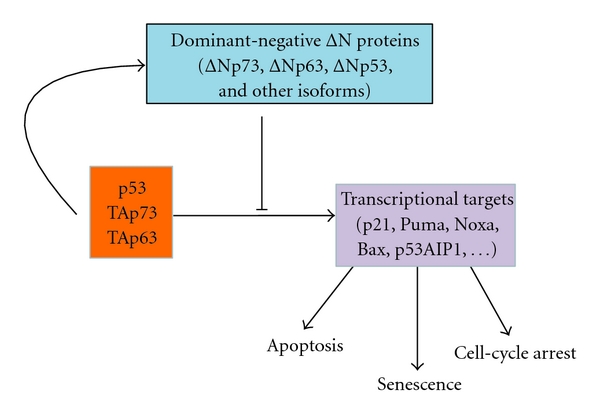
Interactions of p53 family isoforms. N-terminally truncated isoforms of p53, p73, and p63 play a dominant-negative role inhibiting transcriptional and other biological activities of TA isoforms.

 ΔN isoforms function as dominant-negative inhibitors of TA counterparts (Figure 2). Promoter competition and heterocomplex formation have been suggested to explain this phenomenon [17, 34, 35]. In the promoter competition mechanism, the suggestion is that ΔN competes off TA isoforms from their target gene promoters, thus preventing efficient transcription. In the heterocomplex formation mechanism, ΔN isoforms would inhibit TA by forming hetero-oligomeric complexes. 

 ΔN isoforms of p53 and p73 are regulated by a negative feedback loop mechanism. Analogous mechanism was not described for p63 despite its significant similarity to p73. In a nutshell, TA isoforms are able to induce transcription of ΔN isoforms by activating P2 promoters. The induced ΔN isoforms, in turn, inhibit TA isoforms. A good example of these interactions is an induction of Δ133p53 by p53 [5, 36–38]. Similarly, TAp73 and p53 are important regulators of transcriptions of ΔNp73 [39]. It appears that the balance between ΔN and TA isoforms is finely tuned to regulate the activities of TA isoforms. The net effect of these interactions in a given context appears to be dependent on the TA/ΔN expression ratio. Deregulation of this mechanism may lead to tumor development [40–42]. However, it has become clear that the role of ΔN isoforms is multifaceted. The dominant negative concept cannot explain the complexity of all the interactions attributed to ΔN isoforms. Several studies reported that ΔN isoforms can retain transcription activity through additional transactivation domains.

## 3. Role of p53 Isoforms in Cancer

Although many aspects of p53 biology have been thoroughly investigated, the role and regulation of p53 isoforms remain not well understood. 

 Recent studies suggested that Δ133p53 isoform may play an oncogenic role. Mice overexpressing the Δ122p53 isoform (murine homolog of human Δ133p53) show reduced apoptosis, increased cell proliferation and develop a wide-spectrum of aggressive tumors including lymphoma, osteosarcoma, and other malignant and benign tumors [43]. Another phenotypic characteristic of these mice is elevated cytokine levels in the blood and widespread inflammation in many organs. Interestingly, transgenic expression of another p53 isoform, Δ40p53, does not lead to tumor formation in mice, but is associated with a short life span, cognitive decline, and overt diabetes, suggesting a significant difference between these isoforms [44–46]. 

 Several studies reported an elevated expression of Δ133p53 in tumors (Table 1). In breast tumors, 24 of 30 cases showed an increased expression of Δ133p53, but low or undetectable levels in normal breast tissue [5]. An increase of Δ133p53*α* mRNA was also found in renal cell carcinoma [47]. In colon tumors, progression from colon adenoma to carcinoma is accompanied by an increase of Δ133p53 mRNA. This study suggested that Δ133p53 helps to escape from the senescence barrier during colon tumor progression [48]. Interestingly, the Δ133p53 expression level is associated with the mutation status of p53; colon tumors expressing wildtype p53 had higher levels of Δ133p53 than p53 mutant tumors [48]. In addition to Δ133p53, an increased expression of Δ40p53 was also reported in human melanoma cell lines and primary melanoma isolates [33]. However, not all tumors overexpress Δ133p53. Analysis of squamous carcinoma of the head and neck did not reveal any significant changes in the Δ133p53 levels, suggesting that this isoform may only play a tumor-promoting role in a subset of tissues [49].

 Alterations of p53*β* and p53*γ* isoforms were also reported in different types of cancers (Table 1). An increased expression of p53*β* was found in renal cell carcinoma and in most melanoma cell lines. In renal cell carcinoma, p53*β* expression was associated with tumor progression [47]. p53*β* was also found to correlate with worse recurrence-free survival in ovarian cancer patients with functionally active p53 [28]. Decreased p53*β* and p53*γ* mRNA levels were reported in breast cancer [5]. In breast tumors, p53*β* is associated with the expression of estrogen receptor but not with disease outcome [50]. Breast cancer patients expressing both mutant p53 and p53*γ* have lower cancer recurrence and favorable prognosis [51]. Currently, specific functions of p53*β* and p53*γ* remain unclear. A significant hurdle to the studies of p53 isoforms in tumors is the lack of isoform-specific antibodies. The generation of new antibodies, animal models, and additional tumor studies may help to better understand the role of p53 isoforms in tumorigenesis.

## 4. Role of p73 Isoforms in Cancer 

The role of p73 in tumorigenesis is still a matter of debate. In contrast to p53, p73 is rarely mutated and frequently overexpressed in human tumors [23, 52–56]. An initial study of p73-deficient mice found a number of developmental defects and no spontaneous tumors [57]. Follow-up studies have revealed spontaneous tumorigenesis, although the late onset of tumors and smaller tumor sizes compared to p53-deficient animals were reported. The basis for these conflicting results in cancer susceptibility remains obscure but might be related to the animal genetic background and housing conditions. Mice with isoform-specific knockouts of p73 have also been generated; phenotypes of these animals generally reflect previously reported differences between p73 isoforms. TAp73 null mice are tumor prone while ΔNp73 knockouts have increased sensitivity to DNA-damaging agents and elevated p53-dependent apoptosis [58, 59].

 Several studies have found that N-terminally truncated isoforms of p73 play an oncogenic role and are linked to cancer development (Table 1). Targeted transgenic overexpression of human ΔEx2/3p73 in the mouse liver resulted in the development of hepatocellular carcinoma [60]. The N-terminally truncated isoforms are upregulated in many human cancers including liver, ovarian, breast, vulvar cancers, and melanoma [23, 61–68]. Overexpression of ΔEx2p73 and ΔEx2/3p73 was found to be associated with metastases in melanoma [68]. 

 ΔNp73, which is produced by the P2 promoter, has also been found to behave as an oncogene. ΔNp73 facilitates immortalization of primary mouse embryonic fibroblasts and cooperates with oncogenic Ras in their transformation. These transformed cells produce tumors following a subcutaneous injection into nude mice [121, 122]. ΔNp73 also inhibits differentiation of myoblasts and protects them against apoptosis [123]. Studies by others and us found that ΔNp73 is upregulated in a number of tumors and is associated with metastases, chemotherapeutic failure, and poorer patient prognosis [62, 74, 96, 124–130]. 

An important question is what causes deregulation of p73 isoforms in tumors? One of the mechanisms is tumor-specific alternative mRNA splicing. It has been demonstrated that the alternative splicing causes incorporation of a new exon 3' into TAp73 transcripts resulting in a translational switch from TAp73 to ∆Np73 isoform [18, 61]. An interesting observation was also made in hepatocellular carcinoma where an aberrant switch from TAp73 to ΔEx2p73 was found to be mediated by the activation EGFR by amphiregulin. This leads to activation of JNK1 kinase, suppression of splicing factor Slu7, and alternative splicing of p73 transcripts [65]. Activated Ras has also been shown to decrease TAp73 levels and increase ΔNp73 expression during cellular transformation [131]. Abnormal regulation of the P2 promoter has also been reported. We found that transcriptional repressor HIC1 (Hypermethylated In Cancer 1) can suppress expression of ΔNp73 by inhibiting the P2 promoter in normal cells. Loss of HIC1 in esophagus and gastric cancer cells leads to up-regulation of ΔNp73 [96]. In a subset of tumors, abnormal epigenetic changes cause deregulation of p73 isoforms [132–134]. Hypomethylation of the P2 promoter was found in more than half of non-small lung cancers [76]. 

**Figure 3:**
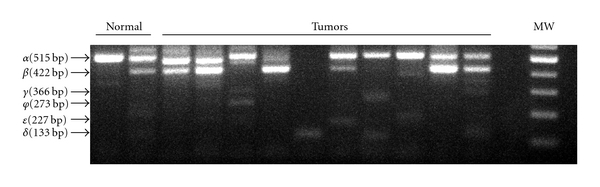
An increased diversity of alternatively spliced species of p73 in colon adenocarcinoma. p73 gene transcription was analyzed in 10 colon tumors and normal colonic mucosa by RT-PCR. Normal specimen 2 represents 14 pooled normal samples. For details, see Vilgelm et al. [15].

 An increased expression of TAp73 isoforms was also found in tumors, although its role remains unclear (Table 1). Several studies suggested that in specific circumstances TAp73 might play a tumor-promoting role [30, 135]. Interestingly, some tumors tend to increase a variety of p73 splice isoforms (Figure 3). In the normal colon and breast, p73*α* and p73*β* isoforms are predominant whereas other spliced variants (*γ*, *δ*, *ϕ*, and *ε*) are primarily detected in colon and breast cancers [15, 23]. This phenomenon was also observed in acute myeloid leukemia. Moreover, the p73*ε* isoform was only expressed in leukemic cells and completely absent in mature myeloid cells [136]. It is currently unclear what role these changes play in tumorigenesis.

## 5. Role of p63 Isoforms in Cancer

Similar to p73, mutations in the p63 gene are rare in human cancers [90, 137, 138]. Several studies reported that ΔNp63 has oncogenic properties. Ectopic overexpression of ΔNp63 in Rat-1A cells promotes colony formation in soft agar. When xenografted into immunocompromised mice, these cells formed tumors [139]. ΔNp63*α* inhibits oncogene-induced cellular senescence and cooperates with Ras to promote tumor-initiating stem-like proliferation [140]. Analysis of p63-deficient mice led to conflicting results with regard to the p63 role in tumorigenesis. p63^−/−^ null mice showed striking developmental defects demonstrating a critical role of p63 in epithelial development [141, 142]. p63^+/-^ heterozygous mice were shown to be susceptible to tumor development [143]. However, other mouse models were not consistent with this observation. Conflicting phenotypes of TAp63 and ΔNp63 transgenic mice have also been reported [144, 145]. 

 ΔNp63 is a predominant isoform expressed in most epithelial cells. Overexpression of ΔNp63 is found in cancers of nasopharyngeal, head and neck, urinary tract, lung, and ovarian tumors and correlated with poor outcome [78, 146–149]. In metastases, ΔNp63 expression was found to be reduced or lost [91, 101]. Microarray analyses revealed the up-regulation of genes associated with tumor invasion and metastasis in p63-deficient cells [150]. It was also reported that p63 suppresses the TGF*β*-dependent cell migration, invasion, and metastasis [151]. This suggests that ΔNp63 plays a dual role by promoting tumor development but suppressing metastases [151, 152]. Expression of ΔNp63 was found to be associated with an increased chemoresistance in a subset of breast and head and neck tumors [153, 154]. 

 TAp63 isoforms induce cellular senescence and inhibit cell proliferation [155–157]. TAp63 deficiency increases proliferation and enhances Ras-mediated oncogenesis [155]. Decreased TAp63 expression is associated with metastasis in bladder and breast cancers as well as poor outcome [42, 90, 158]. TAp63 impedes the metastatic potential of epithelial tumors by controlling the expression of a crucial set of metastasis suppressor genes [151, 159]. 

 Clearly, additional studies are needed to understand the complex regulation of p63 isoforms.

## 6. Interplay of p53/p63/p73 Isoforms in Human Cancers

Interactions between members of the p53 family and their isoforms have a profound effect on tumorigenesis and anticancer drug response. Perhaps, the most studied are interactions between ΔN and TA isoforms. Inhibition of TAp73 by ΔNp63 has been shown to negatively affect the response to platinum-based chemotherapy in head and neck squamous cell carcinomas and a subset of breast tumors [153, 154]. In carcinomas of ovary and childhood acute lymphoblastic leukemia, increased expression of dominant-negative p73 isoforms correlates with resistance to conventional chemotherapy [129, 130]. Moreover, ΔNp73 is primarily expressed in ovarian tumors, which express wildtype p53 [64]. However, crosstalk between the p53 family members is not limited to dominant-negative interactions. Accumulating evidence suggests that the p53 family interacts on multiple levels comprising protein-protein interactions between multiple p53, p63, and p73 isoforms, shared regulation of target genes as well as *TP53* and *TP73* gene promoters [160–163]. In addition, mutant p53 can affect activities of TAp73 and TAp63. It has been shown that certain tumor-derived p53 mutants (R175H, R248W, Y220C, R249S, R283H, and D281G) can physically associate and inhibit activation of TAp73 and/or TAp63 [164–166].

 Current analyses suggest that the function of a particular isoform needs to be investigated in the context of expression of other isoforms. For example, ΔNp73*β* inhibits p53-dependent apoptosis in primary sympathetic neurons [167], but when overexpressed in cancer cells, ΔNp73*β* induces cell cycle arrest and apoptosis [168]. 

 An interesting observation has been made in mouse embryonic fibroblasts, where the combined loss of p73 and p63 results in the failure of p53 to induce apoptosis in response to DNA damage [169]. More recent studies have reported that the p53 family members can simultaneously co-occupy the promoters of p53 target genes and regulate their transcription [15, 170, 171]. Notably, the integral activity of the entire p53 family, as measured by reporter analysis, is a better predictor of chemotherapeutic drug response than p53 status alone [15].

## 7. Conclusion

The p53 family plays a pivotal role in the control of many critical cellular functions. In recent years, it has been revealed that all members of the p53 family are expressed as a diverse variety of isoforms. We only just started to uncover the mechanisms that regulate this diversity. A number of studies also provided the first glimpses of their functional significance. Clearly, isoforms add a new level of functional regulation to many critical biological processes including cell death, proliferation, cell cycle control, and tumorigenesis. Depending on the isoform expressed, the role of a gene can dramatically change from a tumor suppressor to an oncogene. It is also clear that p53, p73, and p63 isoforms tightly interact. A better understanding of this interacting network and its regulation holds the key to future therapeutic benefits.

## Figures and Tables

**Table 1:** Summary of alterations of the p53 family members in human cancers.

Protein	Cancer type	Number of cases	Ref.
	Breast cancer		

p53	(i) p53*β* was detected in 36% breast tumors and associated with the expression of estrogen receptor (ER).	127 breast tumors	[50]
	(ii) p53*γ* was detected in 37% breast tumors and associated with mutations in the p53 gene.		
	(iii) Patients with mutant p53 and p53*γ* isoform had a low cancer recurrence and an overall survival as good as that of patients with wild type p53.		
			
			
	(i) p53, p53*β*, and p53*γ* mRNA, but not transcripts for Δ133p53*α*, Δ133p53*β* mRNA, and Δ133p53*γ*, were detected in normal breast tissues.	30 breast tumors and 8 normal breast samples	[5]
	(ii) p53*β* mRNA was detected in 10/30 tumors; Δ133p53*α* mRNA was detected in 24/30 tumors; p53*γ*, Δ133p53*β*, and Δ133p53*γ* were undetected in tumors.		
	(iii) Some tumors can express mutant p53 but wild type Δ133p53.		

p73	(i) ΔTAp73 and TAp73 mRNA were upregulated in tumors.	60 breast cancers	[62]
	(ii) Expression of ΔEx2p73 (*P* = .05) is associated with vascular invasion; a trend was found between ΔNp73 and vascular invasion (*P* = .06).		
	(iii) Increased expression of ΔEx2p73 and ΔEx2/3p73 were associated with ER status (*P* = .06 and *P* = .07); overexpression of TAp73 was associated with progesterone receptor expression (*P* = .06).		
			
			
	(i) Mutational analysis revealed five silent mutations in 29 hereditary tumors; no p73 mutations were detected in 48 sporadic cancers.	29 hereditary and 48 sporadic breast cancers	[52]
			
			
	(i) Thirteen percent of informative cases showed LOH of the p73 gene; no correlation was found between the p73 LOH and clinical features.	87 primary breast cancer specimens	[69]
	(ii) No changes of p73 transcript levels in breast cancers compared to normal breast tissues.		
	(iii) PCR-SSCP analysis did not detect any missense or frameshift mutations in the p73 gene.		
			
			
	(i) Elevated expression of p73 mRNA was found in 29/77 breast tumors; no correlation of p73 expression with the p53 status.	77 invasive breast cancers	[23]
	(ii) New p73 isoforms were identified.		
	(iii) No coding mutations were found in all coding exons.		

p63	(i) p63 protein was strongly expressed in 13/15 metaplastic carcinomas.	189 invasive breast carcinomas	[70]
	(ii) All metaplastic carcinomas with spindle cells and/or squamous differentiation were positive for p63. One tumor out of 174 nonmetaplastic invasive carcinomas expressed p63.		
			
			
	(i) p63 protein expression was correlated with EBNA-1 immunostaining, suggesting a potential involvement of p63 in mammary tumorigenesis associated with Epstein-Barr virus infection.	85 breast carcinomas	[71]
			
			
	(i) Survival analysis revealed a better prognosis for ER-positive patients with p63 mRNA expression; no other correlations were found.	2,158 ER positive breast cancers and 140 normal breast biopsies.	[72]

	Lung cancer		

p73	(i) p73 mRNA expression was increased in 87% (52/60) tumors compared to normal lung tissues; no correlation with the p53 status was found.	60 lung cancers	[73]
	(ii) No p73 gene amplification was detected.		
	(iii) p73 expression correlated with cancer histology and patient age.		
			
	(i) ΔNp73 expression was detected in the cytoplasm of tumor cells in 77/132 patients with lung cancer. No expression was found in the surrounding normal stromal cells. The expression of ΔNp73 was 52.2%, 50.0%, and 70.2% in stage I, II, and III tumor patients, respectively.	132 lung cancers	[74]
	(ii) ΔNp73 expression was a significant independent factor for predicting poor prognosis.		
			
			
	(i) ΔNp73 protein had primarily nuclear expression in 35/40 cases.	41 NSCLCs	[75]
	(ii) TAp73 protein was found in the cytoplasm in 28/40 cases.		
	(iii) ΔNp73 expression significantly correlated with p53 expression.		
	(iv) No methylation of the P1 promoter was found; P2 promoter was methylated in 17/41 tumors and partially or totally unmethylated in 24/41 cases.		
			
			
	(i) Hypermethylation of the P1 promoter of the p73 gene was relatively uncommon.	102 NSCLCs	[76]
	(ii) Hypomethylation of the P2 promoter was frequently found in squamous cell carcinomas.		
			
	(i) Expression of ΔEx2p73 and ΔEx2/3p73 was increased; expression of ΔNp73 and ΔN'p73 was decreased.	46 NSCLCs	[42]
	(ii) Expression of p73 isoforms correlated with clinicopathological variables.		

p63	(i) p63 protein expression was detected in 109/118 squamous cell carcinomas, 15/95 adenocarcinomas, 2/2 adenosquamous carcinomas, 4/6 large cell carcinomas, 9/20 poorly differentiated neuroendocrine tumors, and 1/37 typical and atypical carcinoids.	221 NSCLCs, 57 stage I–IV neuroendocrine tumors	[77]
	(ii) p63 expression was progressively increased from preneoplastic and preinvasive lesions to invasive squamous cell carcinomas.		
	(iii) p63 immunoreactivity was correlated with the KI-67 labeling index and inversely correlated with the tumor grade in squamous cell carcinomas.		
			
			
	(i) p63 genomic sequence was amplified in 88% of squamous carcinomas, in 42% of large cell carcinomas, and in 11% of adenocarcinomas of the lung. Genomic amplification of p63 is an early event in the development of squamous carcinoma.	217 NSCLCs	[78]
	(ii) ΔNp63*α* was found to be the predominant p63 isoform in normal bronchus and squamous carcinomas but not in normal lung or in adenocarcinomas.		
	(iii) p63 genomic amplification and protein staining intensity were associated with better survival.		
			
			
	(i) p63 protein immunopositivity was found in 80% (48/60) NLCLCs.	60 NSCLCs	[79]
	(ii) Expression of p63 protein was associated with lymph node metastasis and histological classification.		
	(iii) Expressions of p63 and p73 proteins were positively correlated.		
			
			
	(i) Nuclear ΔNp63 staining was found in 77/161 specimens.	161 squamous cell carcinomas	[80]
	(ii) No significant correlation was observed between ΔNp63 expression and clinicopathological variables.		
			
			
	(i) Most of the p63 expression detected in nonneoplastic lung tissue was localized to the nuclei of the bronchiolar basal cells. Nucleic and cytoplasmic expression of p63 protein was found in 46/92 (50%) and 47/92 (51%) cases. Nuclear localization of p63 was correlated with nuclear accumulation of p53, but was not associated with patient survival.	92 lung adenocarcinomas	[81]
	(ii) Cytoplasmic expression of p63 was found to be an adverse prognostic factor in patients with lung adenocarcinoma.		
			
			
	(i) ΔNp63 isoform was upregulated (*P* = .02), and TAp63 was slightly downregulated (*P* = .01).	46 NSCLCs	[42]
	(ii) TAp63 expression correlated with patient survival in non-squamous tumors.		

	Prostate cancer		

p73	(i) No tumor-specific mutations were found in the p73 gene.	27 prostate cancers and 4 prostate cell lines	[82]
	(ii) p73 was biallelically expressed in both normal prostate and tumor tissues.		
	(iii) p73 mRNA expression was not altered in tumors compared to normal prostate.		
			
			
	(i) Significant increase of ΔNp73 mRNA was found in 20/33 (60%) prostate carcinomas and 17/24 (70%) benign prostate hyperplasias. ΔNp73 mRNA was not detected in the normal prostate. None of the specimen expressed ΔN′p73.	33 prostate carcinomas, 24 benign prostatic hyperplasia samples, and 5 normal samples	[83]
	(ii) ΔNp73 expression was significantly associated with the Gleason score. No correlation was found between TAp73 expression and clinical variables.		

p63	(i) p63 expression was reduced in prostate carcinomas compared to matched normal tissues.	20 tumors, 20 metastases, 28 xenografts, and 7 prostate cancer cell lines	[84]
	(ii) One tumor patient had a somatic mutation in exon 11, one prostate cell line, CWR22Rv1, expressed mutant p63 (G to T substitution in exon 8).		
			
	(i) Increased expression of cytoplasmic p63 proteins was associated with increased cancer mortality. Cytoplasmic expression was also associated with reduced levels of apoptosis and increased cellular proliferation.	298 prostate cancers	[85]

	Colon cancer		

p53	(i) Colon adenomas with senescence phenotype expressed elevated levels of p53*β* and reduced levels of Δ133p53. Colon carcinoma tissues were characterized by increased Δ133p53 expression. Colon carcinomas (stage I and II) had increased levels of p53*β* mRNA.	29 colon carcinomas, 8 adenomas, and 9 normal colon specimens	[48]

p73	(i) p73 protein levels were significantly higher in primary colorectal carcinomas.	56 colon carcinomas with matched normal specimens	[86]
	(ii) p73 and VEGF expression levels were correlated (*P* = .016); p73 positive colorectal adenocarcinoma showed significantly greater vascularity.		
	(iii) There were no associations between p73 immunostaining and tumor stage or differentiation.		
			
	(i) TAp73 and ΔTAp73 were significantly co-upregulated in colon cancers.	113 colon cancers	[62]
	(ii) Expression of ΔEx2/3p73 and ΔNp73 isoforms was associated with tumor stage (*P* = .03; *P* = .011).		
	(iii) ΔNp73 overexpression was significantly associated with vascular invasion (*P* = .02).		
	(iv) High levels of ΔEx2/3p73 were associated with lymph node metastases (*P* = .04).		
	(v) Up-regulation of TAp73 was associated with tumor localization (*P* = .004).		
	(vi) Negative p53 staining correlated with overexpression of ΔEx2p73 and TAp73 (*P* = .05; *P* = .05).		

p63	(i) p63 protein was primarily expressed in villous adenomas and poorly differentiated adenocarcinomas.	30 colon adenomas, 30 adenocarcinomas	[87]
	(ii) p63 expression was not associated with p53.		

	Bladder cancer		

p73	(i) p73 mRNA was increased in 18/45 bladder carcinomas and showed a strong correlation with tumor stage or grade; no allelic loss was found. High p73 expression was observed in 4/18 (22.2%), 5/14 (35.7%), and 9/13 (69.2%) of grade I, II, and III tumors, respectively.	45 primary bladder carcinomas	[88]
	(ii) No p73 gene mutations were found by SSCP analysis.		
	(iii) No relationship between p73 and p53 mutations, expression of p21 and MDM2 was found.		
			
	(i) p73 mRNA was increased in 22/23 bladder cancers.	23 primary invasive bladder cancers with matched normal tissues, 7 bladder cancer cell lines	[55]
	(ii) No tumor-specific mutations were found in coding exons of the p73 gene.		
	(iii) p73 was biallelically expressed in the normal bladder and cancer tissues.		
			
			
	(i) p73 protein was undetectable or low in 104/154 (68%) transitional cell carcinomas of the bladder, primarily in invasive tumors.	154 bladder transitional cell carcinomas	[89]
	(ii) Expression of p73 was associated with bladder cancer progression.		

p63	(i) TAp63 was reduced in 25/47 (53.2%) bladder carcinomas. The downregulation of TAp63 was associated with tumor stage and grade.	47 bladder carcinomas and 12 normal specimens	[90]
	(ii) ΔNp63 was increased in 30/47 (63.8%) tumors.		
	(iii) No mutations of p63 gene were found.		
	(iv) No association between p63 expression and the mutational status of p53 or expression of p21Waf1, MDM2, and 14-3-3*σ* in carcinomas was found.		
			
			
	(i) p63 immunostaining was decreased along tumor progression. Basal and intermediate cell layers of normal urothelium showed intense nuclear p63 staining. Lower p63 expression was significantly associated with TNM stage, lymph-node metastasis, and poor prognosis.	75 tumors	[91]
			
			
	(i) ΔNp63 protein expression was increased in tumors and undetectable in normal bladder urothelium. ΔNp63 expression was associated with an aggressive clinical course and poor prognosis. Patients with ΔNp63-negative tumors had a higher recurrence rate than those with ΔNp63-positive tumors.	202 bladder carcinomas and 10 normal specimens	[92]
	(ii) p63*α* expression was decreased in bladder carcinomas.		

	Melanoma		

p53	(i) p53*β* and Δ40p53 mRNAs were expressed in the majority of melanoma cell lines. These isoforms were absent or expressed at low levels in fibroblasts and melanocytes. Δ40p53 was found to inhibit p53-dependent transcription whereas p53*β* enhances it.	19 melanoma cell lines	[33]

p73	(i) p73 mRNA expressed in the majority of human melanoma cell lines, melanocytic nevi, primary malignant melanomas, and metastases.	9 cell lines, 17 melanocytic nevi, 17 primary melanomas, and 20 metastases	[93]
	(ii) No mutation was found in the DNA-binding domain of p73 in 9 melanoma cell lines and 5 metastatic tumors.		
			
			
	(i) ΔEx2p73 and ΔEx2/3p73 mRNAs were significantly upregulated in melanoma metastases.	8 benign melanocytic nevi, 8 primary melanomas, and 19 melanoma metastases	[68]
	(ii) ΔNp73 was the predominant isoform in benign nevi.		
	(iii) An increased expression of ΔEx2p73 and ΔEx2/3p73 isoforms correlated with high levels of TAp73 and E2F1.		

	Gastric cancer		

p73	(i) p73 expression was increased in 37/39 gastric carcinomas and 14/16 matched sets.	39 gastric carcinomas	[94]
	(ii) No allelic deletions or mutations in the p73 gene were detected.		
	(iii) There was no association between p73 expression and mutational status of p53 or expression of p21/Waf1.		
			
			
	(i) p73 expression was found in 33/68 tumors from 24 patients with multiple simultaneous gastric cancers.	68 gastric carcinomas from 32 patients	[95]
	(ii) No mutation in the DNA-binding domain of p73 was found.		
	(iii) No correlations were found between p73 expression and clinical variables.		
			
			
	(i) ΔNp73 mRNA and protein were increased in gastric tumors.	185 tumors	[96]
	(ii) Up-regulation of ΔNp73 protein was significantly associated with poor patient survival. The median survival time for patients with increased ΔNp73 was 20 months whereas that of patients with a negative/weak expression was 47 months.		

p63	(i) p63 expression was found in 25/68 tumors from 24 patients with multiple simultaneous gastric cancer. p63 expression was significantly higher in high-grade diffuse tumors. An increased expression of p63 was observed in intestinal metaplasia and atrophic gastritis. Nonneoplastic tissues had low levels of p63.	68 gastric carcinomas from 32 patients	[95]
	(ii) Expression of TAp63 and ΔNp63 was not associated with the mutational status of p53, tumor stage, or prognosis.		

	Esophageal Cancer		

p73	(i) Low expression of p73 mRNA in 8 analyzed tumors.	48 esophageal tumors (47 ESCCs and 1 EA)	[56]
	(ii) No tumor-specific mutation was found.		
	(iii) LOH for p73 was found in 2/25 (8%) tumors.		
			
			
	(i) LOH was found in 9/14 cases.	15 ESCCs	[97]
	(ii) No mutations in the p73 gene were detected in tumor samples. A polymorphism at codon 173 of p73 was identified.		
	(iii) p73 mRNA was overexpressed in 9/15 tumor samples. Four cases showed loss of imprinting. Expression of p73 correlated with p53 mutations.		
	(i) p73 immunoreactivity was reduced with cancer invasion.	106 esophageal cancers	[98]
	(ii) No associations were found between p73 expression and clinicopathological variables.		
	(iii) Inverse correlation between p73 expression and p53 status was found. Expression of p21 correlated with the p73 expression.		
			
	(i) Expression of ΔNp73 mRNA and protein was increased in esophageal adenocarcinoma.	68 EA and GEJ tumors	[96]
	(ii) HIC (hypermethylated in tumors 1) protein, but not p53, was found to regulate ΔNp73.		
	(iii) Expression of ΔNp73 significantly correlated with the expression of TAp73.		

p63	(i) p63 protein was diffusely expressed in all cases of esophageal squamous cell dysplasia and carcinoma.	20 normal esophageal squamous tissues, 4 squamous dysplasias, 7 squamous cell carcinomas, 10 BE, 13 BE-associated multilayered epithelial specimens, 10 esophageal mucosal gland duct specimens, 12 BE-associated dysplasias, and 7 BE-associated adenocarcinomas	[99]
	(ii) No expression was found in all cases of esophageal adenocarcinoma and Barrett's esophagus.		
	(iii) ΔNp63 mRNA was a predominant isoform in all benign and neoplastic squamous tissues.		
			
			
	(i) p63 expression was restricted to the basal cell layer in normal esophageal epithelium. Strong expression of p63 was frequent finding in squamous precancerous and cancerous lesions. BE-derived lesions expressed p63 at low levels.	50 esophageal adenocarcinomas, 41 adjacent specialized metaplastic epithelium, 27 low-grade intraepithelial neoplasias, and 21 high-grade intraepithelial neoplasias, 50 ESCCs, 4 squamous low-grade intraepithelial neoplasias, and 18 squamous high-grade intraepithelial neoplasias	[100]
	(ii) p63 gene amplification was found to be infrequent in esophageal malignancies. p63 gene amplification was found in 2/10 squamous cell carcinomas and in 1/10 adenocarcinomas.		
			
			
	(i) ΔNp63 protein was expressed in 32% and 64% carcinomas with and without adventitial invasion, and in 37% and 65% with and without lymph node metastasis, respectively. A better prognosis was observed in patients with ΔNp63 expression.	61 ESCCs	[101]
	(ii) ΔNp63 expression was associated with patient survival. Decreased expression of p63 was more frequent in advanced carcinomas.		
			
			
	(i) p63 expressed in 171/180 (95%) patients.	180 ESCCs	[102]
	(ii) Patients with p63-positive tumors had better overall survival compared to patients with p63-negative tumors.		
	(iii) Correlation between p63 and clinicopathological parameters was not significant. Negative p63 expression tended to correlate with distant metastases and clinical stage.		
			
			
	(i) Expression of p63 protein was increased in tumors. It was detected in 21/40 (52.5%) ESCCs.	40 ESCCs and 40 normal esophageal specimens	[103]
	(ii) No associations were observed between expression of p63 protein and clinicopathological variables.		

	Head and neck cancer		

p53	(i) p53*β* mRNA was detected in 18/20 tumor specimens (T), 13/14 normal tissues adjacent to the tumor (N), and 6/6 normal control specimens (NS); p53*γ* was detected in 5/20 (T), 3/14 (N), and 6/6 (NS); ∆133p53*α* expressed in 7/20 (T), 9/14 (N), and 3/14 (NS); Δ133p53*β* was detected in 3/20 (T), 2/14 (N); Δ133p53*γ* expressed in 4/20 (T), 1/14 (N), 2/6 (NS).	21 squamous cell carcinomas, 16 normal specimens adjacent to tumors, 8 normal specimens	[49]

p73	(i) Two missense mutations at codons 469 and 477 and one silent mutation at codon 349 in the p73 gene were found.	67 primary oral and laryngeal squamous cell carcinomas	[104]
	(ii) Increased p73 expression was found in 5/21 (23.8%) patients; decreased expression was observed in 6/21(28.5%) patients.		
			
			
	(i) p73 mRNA was decreased in 5/17 (30%) tumors. No mutation and LOH was found in the p73 gene.	50 squamous cell carcinomas	[105]
	(ii) No correlation was found between p73 and p53 protein expression.		
			
	(i) p73 protein expression was detected in 12/68 (18%) normal mucosas and 32/68 (47%) HNSCC.	68 squamous cell carcinomas	[106]
	(ii) No p73 mutations were found in primary and recurrent carcinomas.		
	(iii) No correlation was found between protein expression of p73 and p53.		
			
			
	(i) p73 was significantly elevated in buccal epithelial dysplasia (protein) and squamous cell carcinomas (protein and mRNA) compared to normal control tissues.	25 buccal squamous cell carcinomas, 75 epithelial dysplasias	[107]
	(ii) p73 expression was associated with cervical lymph node metastasis for cases of buccal SCC.		

p63	(i) Positive immunostaining for p63 was detected in 55/68 (81%) carcinomas, 40/68 (59%) normal tissues.	68 squamous cell carcinomas	[106]
	(ii) No p63 mutations were detected in primary and recurrent carcinomas.		
	(iii) No correlation was found between p63 and p53 protein expression.		
			
			
	(i) Expression of p63 was associated with tumor differentiation. p63 expression was increased in poorly differentiated tumors.	96 oral squamous cell carcinomas and 10 normal specimens	[108]
	(ii) Increased p63 expression was associated with poor patient survival. No significant correlations were found between p63 expression and sex, age, tumor size, staging, recurrence, and metastasis. Tumors with diffuse p63 expression were more aggressive and poorly differentiated.		

	Cervical cancer		

p73	(i) ΔNp73 and TAp73*α* proteins were overexpressed in tumors.	117 cervical squamous cell carcinomas and 113 normal specimens	[109]
	(ii) The overexpression of ΔNp73 was correlated with the resistance to radiation therapy. An increased expression of TAp73*α* was detected in the majority of cervical squamous cell carcinomas sensitive to irradiation.		
	(iii) ΔNp73 expression was associated with recurrence of the disease and an adverse outcome. TAp73*α* predicted a better survival.		
			
	(i) Higher TAp73 expression was found in high-grade lesions and carcinomas (*P* < .0001).	91 high-grade and 107 low-grade squamous intraepithelial lesions, 212 ASC-US, 56 squamous cell carcinomas, and 63 normal specimens	[110]
	(ii) No correlation was found between p73 and p63 immunostainings.		

p63	(i) Expression of p63 protein was high in 97% squamous cell carcinomas. p63 is a strong marker for squamous differentiation.	250 cervical carcinomas	[111]
	(ii) Transitions from squamous to columnar or undifferentiated tumors coincided with the loss of p63 expression.		
	(iii) HPV16 positivity and p63 expression were strong associated.		
	(i) ΔNp63 staining was increased with tumor progression. All SCCs, transitional cell carcinomas, and adenoid basal carcinomas were positive for p63.	127 uterine cervical tissues with various lesions	[112]
	(ii) ΔNp63 protein was undetected in all adenocarcinomas.		
			
	(i) Increased p63 immunostaining was found in high-grade lesions and cervical carcinomas.	91 high-grade and 107 low-grade squamous intraepithelial lesions, 212 ASC-US, 56 squamous cell carcinomas, and 63 normal specimens	[110]
	(ii) Significant correlation was found between the presence of high-risk HPV and p63 expression.		
	(iii) No correlation was found between p63 and p73 immunostainings.		

	Renal cancer		

p53	(i) All six p53 isoforms were detected in tumor and normal tissues with the exception of Δ133p53*β*, which was not detected in normal tissues.	41 renal cell carcinomas and normal tissues adjacent to tumor	[47]
	(ii) p53*β* mRNA was significantly upregulated in tumor samples (*P* < .001) and associated with tumor stage.		

p73	(i) Monoallelic expression of p73 was found in 11/12 normal tissues; biallelic expression in 8/12 cancers.	28 renal cell carcinomas	[113]

p63	(i) p63 expression was detected in 25/27 (92.6%) urothelial carcinomas. None of the studied renal cell carcinomas was positive for p63. p63 expression correlated with tumor stage, grade and survival time, but not with the tumor progression.	42 renal cell carcinomas and 27 renal pelvis urothelial carcinomas	[114]

	Thyroid cancer		

p73	(i) p73 transcripts were downregulated in adenomas and differentiated carcinomas.	102 thyroid tissues from 60 patients	[115]
	(ii) Expression of TAp73 and ΔNp73 transcripts correlated with expression of p53, p14ARF, and p16INK4a mRNA in normal tissue. These correlations were lost in carcinomas.		
			
			
	(i) ΔNp73 was expressed in 27.3% follicular adenomas, 85.4% follicular carcinomas, 99.2% papillary carcinomas, and 95.7% anaplastic carcinomas. Normal follicular cells were negative for ΔNp73 protein. In papillary carcinoma, ΔNp73 levels were inversely correlated with tumor size, extrathyroid extensions, and metastases. In anaplastic carcinoma, ΔNp73 expression was significantly lower than in papillary carcinoma.	223 thyroid neoplasms	[116]

p63	(i) TAp63*α* protein was expressed in 25/27 thyroid cancers 1/7 benign adenomas, but not in normal thyroid (0/8). TAp63*α* transcripts, but not TAp63*β*, TAp63*γ*, and ΔNp63, were expressed in tumors. Thyroid cancer cell lines also expressed p63.	27 thyroid cancers, 11 cell lines	[117]

	Pancreatic cancer		

p73	(i) Expression of p73 protein was detected in 45.6% cancers and was primarily found in cystic adenocarcinomas.		[118]
	(ii) p73 expression was inversely correlated with lymph node metastasis, tumor size, and Ki-67 labeling index.		
	(iii) No correlation was found between p73 and p53 protein expression.		
			
			
	(i) p73 methylation was found in more than 50% noninvasive and invasive tumors.	28 intraductal papillary mucinous neoplasms	[119]

p63	(i) Overexpression of p63 protein was observed in 68.2% cancers.		
	(ii) p63 expression was not associated with clinicopathological variables.		[118]
	(iii) No correlation was found between p63 and p53 protein expression.		
			
			
	(i) No ΔNp63 protein expression was found in normal pancreatic ducts and all pancreatic intraepithelial neoplasias. Among invasive carcinomas, ΔNp63 expression was detected only in areas of squamous differentiation and was completely absent in ordinary ductal areas. ΔNp63 is a reliable marker of squamous differentiation in the pancreas. It was valuable in distinguishing squamous/transitional metaplasia from PanINs.	25 nonneoplastic pancreata, 25 pancreatic intraepithelial neoplasia, and 50 pancreatic ductal adenocarcinomas	[120]
